# Case report: Hepatic inflammatory pseudotumor-like follicular dendritic cell sarcoma: A rare case and minireview of the literature

**DOI:** 10.3389/fmed.2022.1002324

**Published:** 2022-11-08

**Authors:** Fan Ding, Chao Wang, Chi Xu, Hui Tang

**Affiliations:** ^1^Center of Gallbladder Disease, East Hospital of Tongji University, Shanghai, China; ^2^Institute of Gallstone Disease, Tongji University School of Medicine, Shanghai, China; ^3^Department of Radiology, Nanxiang Hospital of Jiading District, Shanghai, China; ^4^Department of Hepatic Surgery and Liver Transplantation Center, The Third Affiliated Hospital of Sun Yat-sen University, Guangzhou, China; ^5^Organ Transplantation Institute, Sun Yat-sen University, Guangzhou, China

**Keywords:** inflammatory pseudotumor, follicular dendritic cell, sarcoma, case report, hepatic

## Abstract

Inflammatory pseudotumor (IPT)-like follicular dendritic cell sarcoma (FDCS) is a rare neoplasm referred to as the FDCS variant. Here we report a 66-year-old female patient suffering from hepatic IPT-like FDCS and summarize IPT-like FDCS reported in the literature. The patient presented with obvious abdominal pain without significant laboratory abnormalities and subsequently underwent surgical resection of a hepatic lesion. Postoperative pathological results demonstrated a vascular tissue-rich neoplasm (7.0-cm maximum diameter). The tumor cells expressed CD21 and CD35, and *in situ* hybridization detected Epstein–Barr virus-encoded RNA (EBER). Metastasis or recurrence was not detected during the 7-year follow-up.

## Introduction

Follicular dendritic cells (FDCs) develop from perivascular precursors of stromal cell origin that are essential for the organization and maintenance of lymphoid architecture, induction of the germinal center reaction, production of B memory cells, and protection from autoimmune disorders (1). FDC sarcoma (FDCS) is an extremely rare neoplasm with nearly more than half of the cases occurring in lymph nodes (2). Extranodal FDCS, which mainly arises from intraabdominal organs such as liver, spleen, colon, and pancreas, may display systemic clinical symptoms (3). Inflammatory pseudotumor (IPT)-like FDCS is a recently described unique subtype of FDCS with different histological appearances and behavior compared with those of classical FDCS. The most recent World Health Organization (WHO) classification notes that IPT-like FDCS appears to be indolent; however, the data on clinical outcomes are limited (4).

The cause of IPT-like FDCS is unknown, and the diagnostic criteria are not definitive. However, Epstein–Barr virus (EBV) infection is considered one of the most important etiologies of this sarcoma (4). Distinguishing IPT-like FDCS from other tumors is very challenging, and such tumors are commonly misdiagnosed as inflammatory lesions or other malignant neoplasms. Definite diagnosis of IPT-like FDCS should rely on radiology, cellular morphology, histopathology, and immunochemistry. IPT-like FDCS exhibits indolent features, prognosis is favorable, and surgical excision is the best treatment.

Herein, we report the case of a 66-year-old female with an intraabdominal IPT-like FDCS in the hepatic right lobe. By analyzing the distinctive clinicopathologic features of this rare case combined with reviewing the related literature, here we summarize the current clinical features and diagnosis of IPT-like FDCS and discuss the treatment and prognosis of this tumor subtype.

## Literature review

We systematically searched the PubMed, EMBASE, and MEDLINE databases using the search terms “inflammatory pseudotumor-like” combined with “follicular dendritic cell sarcoma,” or “follicular dendritic cell tumor,” or “fibroblastic dendritic cell sarcoma,” or “fibroblastic dendritic cell tumor” in research published from 2000 to 2022. We collated demographic, clinicopathological, and follow-up information (Table 1).

**TABLE 1:** Clinical characteristics of patients with inflammatory pseudotumor-like follicular dendritic cell sarcoma.

References	Case	Age (y)/ Gender	Location	Main complaints	Maximum diameter (cm)	EBER	Treatment	Follow-up	Recurrence or metastasis
Shi et al. (29)	1	77/F	Colon	Lower abdominal pain with hematochezia	3.0	+	Endoscopic polypectomy	15 months	N
Zhao et al. (30)	2	56/F	Colon	Asymptomatic	3.2	+	Surgery	14 months	N
Xue et al. (31)	3	45/F	Spleen	Waist soreness	2.9	NA	NA	NA	NA
	4	40/F	Spleen	Asymptomatic	7.3	NA	NA	NA	NA
	5	81/M	Spleen	Asymptomatic	8.1	NA	NA	NA	NA
	6	59/F	Spleen	Left upper abdominal pain	15.0	NA	NA	NA	NA
	7	54/F	Spleen	Left upper abdominal pain	3.6	NA	NA	NA	NA
	8	71/F	Spleen	Asymptomatic	4.5	NA	NA	NA	NA
	9	67/M	Spleen	Asymptomatic	6.0	NA	NA	NA	NA
Xu et al. (20)	10	81/M	Liver	Asymptomatic	NA	NA	Resection	12 months	N
	11	53/M	Liver	Abdominal distension	NA	NA	Resection	24 months	N
	12	76/F	Spleen	Asymptomatic	NA	NA	Resection	10 months	N
	13	49/F	Spleen	Asymptomatic	NA	NA	Resection	24 months	N
	14	73/M	Spleen	Asymptomatic	NA	NA	Resection	18 months	N
	15	66/F	Liver and spleen	Epigastric pain	NA	NA	Resection	20 months	N
	16	62/F	Spleen	Asymptomatic	NA	NA	Resection	18 months	N
	17	43/F	Spleen	Abdominal distension	NA	NA	Resection	24 months	N
	18	36/M	Spleen	Fever	NA	NA	Resection	24 months	N
	19	41/F	Spleen	Asymptomatic	NA	NA	Resection	17 months	N
	20	88/M	Spleen	Asymptomatic	NA	NA	Resection	12 months	N
Pascariu et al. (32)	21	34/F	Liver	Epigastric pain	6.0	+	Laparoscopic hepatectomy	2 months after reoperation	72 months after first surgery
Nguyen et al. (33)	22	44/F	Spleen	Left upper quadrant abdominal pain	2.5	+	Laparoscopic splenectomy	NA	12 months after first surgery
Morales-Vargas et al. (34)	23	66/F	Spleen	Left upper quadrant pain	5.0	+	Splenectomy	6 months	N
Lu et al. (35)	24	55/F	Liver	Epigastric pain	14.5	+	Hepatectomy	60 months with PR	Paravertebral metastasis and recurrence
Liu et al. (4)	25	61/M	Liver	Asymptomatic	4.2	–	Laparoscopic hepatectomy	13 months	N
Li et al. (36)	26	47/M	Liver	Right upper quadrant abdominal pain	20.0	+	Hepatectomy	50 months	N
He et al. (37)	27	64/F	Lung	Asymptomatic	2.0	+	Lobectomy	10 months	N
Bruehl et al. (38)	28	70/M	Spleen	Epigastric pain	9.9	+	Splenectomy	24 months	N
Zhuang et al. (8)	29	27/F	Spleen	Desquamative stomatitis	9.0	NA	Splenectomy	12 months	N
Jin et al. (39)	30	38/M	Liver	Asymptomatic	12.4	+	Hepatectomy	NA	NA
Mograbi et al. (40)	31	70/F	Pancreas and spleen	Asymptomatic	7.0	+	Pancreatectomy and splenectomy	NA	NA
Li et al. (19)	32	31/F	Liver	Asymptomatic	3.6	+	Hepatectomy	26 months	N
	33	48/M	Liver	Asymptomatic	10.0	+	Hepatectomy	10 months	N
Wu et al. (21)	34	52/M	Spleen	NA	7.0	+	Resection	84 months	N
	35	46/M	Liver	NA	3.5	+	Resection	35 months	N
	36	37/F	Liver	NA	8.5	+	Resection	14 months	N
	37	64/F	Liver	NA	11.0	+	Resection	41 months	N
	38	63/F	Spleen	NA	4.0	+	Resection	17 months	N
	39	54/F	Spleen	NA	8.5	+	Resection	7 months	N
	40	53/M	Spleen	NA	3.0	+	Resection	3 months	N
Li et al. (41)	41	64/F	Spleen	Epigastric pain	7.2	+	Laparoscopic splenectomy	8 months	N
	42	61/M	Spleen	Asymptomatic	6.2	+	Laparoscopic splenectomy	16 months	N
	43	42/F	Spleen	Left-sided flank pain	4.0	+	Laparoscopic splenectomy	9 months	N
	44	57/F	Spleen and lung	Epigastric pain	13.3	+	Laparoscopic splenectomy	4 months	Pulmonary metastasis
	45	52/M	Spleen and vertebra	Back pain	3.7	+	Laparoscopic splenectomy	5 months	Multiple bone metastasis
Kwon et al. (42)	46	58/F	Spleen	Asymptomatic	5.0	+	Splenectomy	24 months	N
Kazemimood et al. (25)	47	53/F	Colon	Abdominal discomfort	3.0	–	Laparoscopic right colectomy	NA	NA
Hang et al. (15)	48	57/M	Spleen	Asymptomatic	2.7	+	laparoscopic splenectomy	9 months	N
Wang et al. (9)	49	60/F	Left axillary region and neck	Myasthenia	6.4	–	Rituximab	2 months after discharge	Dead due to MODS
Kitamura et al. (43)	50	74/F	Spleen	Asymptomatic	2.9	+	Splenectomy	24 months	N
Hu et al. (44)	51	49/F	Left adrenal gland	Asymptomatic	5.0	NA	Left adrenalectomy	58 months	The tail of pancreas recurrence
Gong et al. (45)	52	42/F	Colon	Asymptomatic	4.5	+	Endoscopic excision	16 months	N
Bui et al. (18)	53	50/F	Spleen	Abdominal pain	6.5	+	Splenectomy	NA	NA
You et al. (46)	54	43/M	Liver	Right upper quadrant pain	20.0	+	Unresectable	NA	NA
Vardas et al. (47)	55	61/M	Spleen	Asymptomatic	10.0	+	Splenectomy	12 months	N
Rao et al. (48)	56	39/M	Spleen	Asymptomatic	7.2	+	NA	NA	NA
Pan et al. (49)	57	78/F	Colon	Bloody stool	3.9	+	Polypectomy	5 months	N
Li et al. (16)	58	49/F	Spleen	Asymptomatic	4.7	+	Splenectomy	NA	NA
	59	56/F	Spleen	Abdominal pain	8.0	+	Splenectomy	17 months	N
	60	38/M	Liver	Fatigue, anorexia	8.5	+	Left lobectomy of liver	11 months	N
	61	42/F	Liver	Abdominal pain	2.0	+	Wedge resection	36 months	N
	62	50/M	Spleen and liver	Abdominal bloating, fatigue	10.0	+	Splenectomy and left lobectomy of liver	17 months	N
	63	39/F	Liver	Asymptomatic	9.0	+	Hepatic lobectomy	84 months after chemotherapy and mass excision	Recurrence at 12 months
Ge et al. (3)	64	54/F	Spleen	left upper quadrant pain	3.5	+	Splenectomy	10 months	N
	65	79/M	Spleen	Epigastric pain	6.0	+	Splenectomy	18 months	N
Kim et al. (50)	66	76/M	Spleen	Asymptomatic	3.2	+	Splenectomy	7 months	N
Choe et al. (13)	67	64/F	Spleen	Asymptomatic	5.5	+	Splenectomy	78 months	N
	68	72/F	Spleen	Asymptomatic	7.2	+	Splenectomy	18 months	N
	69	53/F	Spleen	Asymptomatic	3.2	+	Splenectomy	13 months	N
	70	76/M	Spleen	Asymptomatic	3.2	+	Splenectomy	8 months	N
	71	72/M	Spleen	Asymptomatic	6.0	+	Splenectomy	18 months	N
	72	75/M	Spleen	Abdominal pain	3.5	+	Splenectomy	30 months	N
Takahashi et al. (27)	73	39/M	Spleen	Asymptomatic	7.0	–	Splenectomy	31 months	N
Kiryu et al. (51)	74	56/F	Spleen	Asymptomatic	4.0	+	Splenectomy	24 months	N
	75	60/M	Spleen	Asymptomatic	2.0	+	Splenectomy	48 months	N
	76	78/F	Spleen	Asymptomatic	2.0	+	Splenectomy	48 months	N
Yoon et al. (52)	77	64/F	Spleen	Asymptomatic	5.1	+	Splenectomy	NA	NA
Agaimy et al. (26)	78	52/M	Ileal mesentery	Acute abdomen	6.0	–	Emergency excision	Lost	Lost
Horiguchi et al. (53)	79	77/F	Spleen	Epigastric pain	8.5	+	Splenectomy	36 months	N
Brittig et al. (54)	80	54/M	Spleen	Asymptomatic	12.0	+	Splenectomy	48 months	N
Wu et al. (55)	81	45/M	Liver	Epigastric pain	6.7	+	Right hepatectomy	9 months	N
Zhang et al. (56)	82	31/F	Liver	Anorexia	3.5	+	Laparoscopic right hepatectomy	10 months	N
	83	48/M	Liver	Asymptomatic	10.0	+	Right hepatectomy	2 months	N
Deng et al. (57)	84	67/F	Liver	Cough	4.0	+	Hepatectomy	NA	NA
Ang et al. (58)	85	63/F	Liver	Fever	13.4	+	Right hemihepatectomy	48 months	N
Zhang et al. (59)	86	19/F	Liver	Abdominal discomfort	6.0	+	Hepatic VII segmental resection	12 months	N
Chen et al. (60)	87	28/F	Liver	Abdominal pain	6.0	+	Left lobectomy of liver	NA	Recurrence at 48 months
	88	39/M	Spleen	Asymptomatic	7.4	+	Splenectomy	40 months	N
	89	48/M	Liver	Abdominal pain	23.3	+	Extended right hemihepatectomy	23 months	N
	90	65/M	Spleen and Liver	Epigastric pain	23.3	+	Splenectomy and radical dissection of retroperitoneal lymph nodes	2 months	Dead for cachexia
	91	51/M	Spleen	Malaise, weight loss	8.5	+	Splenectomy	19 months	N
	92	68/M	Spleen	Asymptomatic	2.3	+	Splenectomy	6 months	N
	93	51/F	Spleen	Abdominal discomfort	5.3	+	Splenectomy	5 months	N
	94	67/M	Spleen	Asymptomatic	7.5	+	Splenectomy	5 months	N
	95	60/M	Liver	Asymptomatic	3.0	+	Wedge resection	3 months	N
	96	52/F	Spleen	Asymptomatic	0.9	+	Splenectomy	12 months	N
Nguyen et al. (61)	97	57/F	Liver and Spleen	Weight loss	NA	+	Rejection treatment	NA	NA
Granados et al. (62)	98	57/F	Liver	Abdominal pain	13.0	+	Resection	24 months	N
Cheuk et al. (6)	99	19/F	Liver	Right upper quadrant pain	12.0	+	Resection	40 months	N
	100	56/F	Liver	Abdominal discomfort	15.0	+	Resection of right lobe of liver	56 months	Recurrence at 15, 27, 35 months respectively
	101	40/F	Liver	Epigastric pain	12.5	+	Left hepatectomy	108 months	Recurrence at 108 months
	102	49/F	Liver	Asymptomatic	4.2	+	Resection	9 months	N
	103	37/M	Liver	Weight loss	15.0	+	Right trisegmentectomy	42 months	N
	104	35/F	Liver	Abdominal discomfort	20.0	+	Right hemihepatectomy	95 months	Dead for disseminated tumor
	105	31/F	Liver	Abdominal distension	15.0	+	Right hemihepatectomy	60 months	N
	106	58/F	Spleen	Abdominal discomfort	22.0	+	Splenectomy	4 months	N
	107	39/F	Spleen	Weight loss	7.5	+	Splenectomy	2 months	N
	108	61/F	Spleen	Asymptomatic	3.5	+	Splenectomy	NA	NA
	109	49/F	Peri-pancreas	Abdominal distension	9.5	+	Whipple’s operation	NA	NA
Chen et al. (63)	110	57/F	Liver	Epigastric pain	9.5	+	Refusion surgical resection	36 months	N
	111	51/F	Liver	Abdominal distension	12.0	+	Left lobectomy	12 months	N
Lewis et al. (11)	112	81/F	Spleen	Epigastric pain	5.0	+	Splenectomy	18 months	N
Nishiyama et al. (64)	113	73/F	Spleen	Asymptomatic	8.0	+	Splenectomy	144 months	N
Present case	114	66/F	Liver	Abdominal pain	7.0	+	Hepatic segment VI and VII resection	84 months	N

N, none; NA, not available; MODS, multiple organ dysfunction syndrome.

## Case presentation

In 2015, a 66-year-old woman who suffered from right upper abdominal pain was admitted to the Department of Hepatic Surgery and Liver Transplantation Center at the Third Affiliated Hospital of Sun Yat-sen University because of a liver mass detected using abdominal ultrasound at a local hospital. Based on ultrasound examination and medical history, the preliminary diagnosis was liver-occupying lesions and diabetes mellitus type 2. The patient did not complain of vomiting, nausea, fever, or diarrhea. After admission, the values of routine tests including liver and kidney function and routine blood tests were almost within their normal limits. Liver function according to the Child–Pugh classification was class A. Serological analyses to detect hepatitis virus, syphilis, and human immunodeficiency virus were negative. Furthermore, the levels of common female-specific tumor markers, particularly α-fetoprotein (AFP), carcinoembryonic antigen (CEA), carbohydrate antigen (CA)-199, and CA-125 were normal. The patient had a long history of diabetes mellitus type 2 and achieved good fasting plasma-glycemic control with acarbose combined with metformin.

**FIGURE 1:**
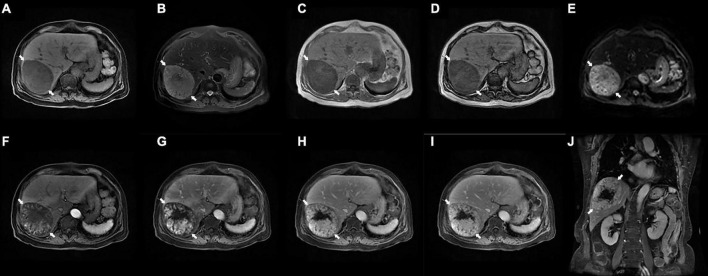
Preoperative enhanced magnetic resonance imaging (MRI) examination. **(A)** T1WI shows an ovalshaped hypointense lesion with clear border in the right lobe of the liver (arrows, 94×74 mm). **(B)** Fat-suppressed T2WI shows a slightly hyperintense lesions (arrows). **(C,D)** The in-phase **(C)** and out-of-phase **(D)** of T1WI demonstrates hypointense speckled signals within the mass (arrows). **(E)** DWI shows a hyperintense lesions (arrows). **(F–J)** Enhanced MRI scans showed progressive enhancement of the lesion (arrows) on the early **(F)** and late **(G)** arterial phase, portal venous phase **(H)**, delayed phase **(I)**, and coronal view **(J)**, while no enhancement was seen in the necrotic region. MRI, magnetic resonance imaging; T1WI, T1 weighted image; T2WI, T2 weighted image; DWI, diffusion weighted imaging.

Preoperative enhanced magnetic resonance imaging (MRI) showed a mass approximately 94 × 74 mm with clear borders in hepatic segments VI and VII. On enhanced phase, the images showed progressive enhancement of the lesion, while enhancement was not seen in the necrotic region. Significantly, the lesion demonstrated slightly hypointense speckled signals on in-phase and out-of-phase T_1_WI. Therefore, the primary diagnosis highly suggested a hepatic fat-poor angioleiomyolipoma (Figure 1).

Although the diagnosis was not definitive, the large liver mass caused significant clinical symptoms (abdominal pain) in the absence of concurrent systematic disease. Our multidisciplinary hepatic surgery team therefore proposed surgical resection as the most appropriate procedure to confirm a diagnosis and further formulate the treatment strategy. Subsequently, the patient underwent resection of hepatic segments VI and VII, and minor complications occurred postoperation. During surgery, lesions were not observed in the gastrointestinal tract, spleen, mesentery, or other abdominal organs. The operation lasted 135 min, and the estimated intraoperative blood loss was approximately 150 mL.

**FIGURE 2:**
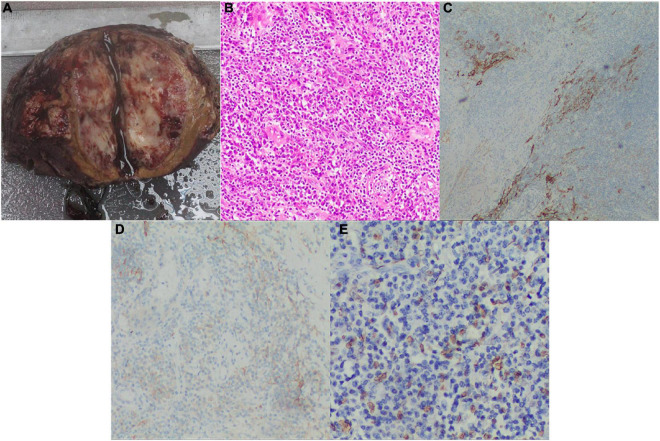
Postoperative pathology examinations. **(A)** Grossly, the cut surface of the fleshy neoplasm with necrosis and hemorrhage (tumor size: 7.0×5.0 cm); **(B)** H&E stained image showing that the tumor tissue had a meshwork-like architecture with abundant vascular-like proliferation, magnification: 200X; **(C)** The positive IHC result of CD21, magnification: 100X; **(D)** The positive IHC result of CD35, magnification: 100X; **(E)** The positive result of EBV for *in situ* hybridization, magnification: 200X. H&E, hematoxylin and eosin; IHC, immunohistochemical; EBV, Epstein Barr virus.

Grossly, the size of the tumor was approximately 7.0 × 5.0 cm, presenting with an indistinct boundary and a patchy gray-red section with intratumor hemorrhage and necrosis. Postoperative pathology showed a sarcoma containing varying sizes of vessel lumens with negative surgical margins. The neoplastic tissue was extensively infiltrated by definite lymphocytes, plasma cells, and spindle cells. The tumor cells were fusiform and ovoid with a translucent cytoplasm and large vacuolated nuclei. According to the infiltration of numerous lymphocytes into the neoplastic tissues and immunohistochemical detection of CD21 and CD35 expression as well as *in situ* hybridization detection of Epstein–Barr virus-encoded RNA (EBER), the morphological and immunophenotypic results were consistent with a diagnosis of IPT-like FDCS (Figure 2). Hence, the final diagnosis was revised to IPT-like FDCS.

The patient was discharged without adjuvant chemotherapy or radiotherapy and has been examined at our hepatic surgery follow-up clinic every 6 months. The outcome of the 7-year follow-up was good, and metastasis or recurrence was not detected.

## Discussion

Follicular dendritic cell sarcoma (FDCS) is a rare mesenchymal tumor of follicular dendritic cell origin originally identified by Monda et al. in 1986 (2). FDCS is classified into the two histopathological subtypes as follows: conventional and inflammatory pseudotumor-like (5). IPT-like FDCS is an extremely rare tumor, and only 113 cases are published (Table 1).

Inflammatory pseudotumor (IPT)-like FDCS possesses morphological and clinical features intermediate between inflammatory pseudotumors and FDC tumors and was first classified in 2001 as a distinct variant (6). Compared with conventional FDCS, IPT-like FDCS exhibits unique histopathological and clinical features that generally occur in abdominal organs, almost exclusively involving the spleen, liver, or both (104/114); and colonic (5/114), mesenteric (2/114), pancreatic (1/114), pulmonary (2/114), paranephric (1/114), and lymphatic (1/114) involvement occur as well.

Inflammatory pseudotumor (IPT)-like FDCS predominantly occurs in middle-aged adults (median age, 54.5 years; range, 19–88 years), with marked female predominance (female to male ratio = 1.71:1). Patients are mainly asymptomatic or present with abdominal distension or pain, occasionally accompanied by systemic symptoms such as back pain, waist soreness, significant weight loss, fever, and weakness. In rare cases, IPT-like FDCS exhibits paraneoplastic arthritis (7), and paraneoplastic pemphigus (8–10).

The pathogenesis and causes of IPT-like FDCS are unknown, although Epstein–Barr virus (EBV) may play an essential role in etiology. Stimulation by EBV may induce FDCs derived from mesenchymal cells to undergo neoplastic transformation and express CD21 and CD35 (11, 12). Interestingly, Choe et al. (13) found remarkable numbers of IgG4-positive plasma cell in 6 Asian patients with EBV-positive IPT-like FDCS of the spleen, suggesting that the intimate relationship between IgG4 and EBV plays a critical role in IPT-like FDCS, which may be limited to Asians. Moreover, mutations in genes encoding components of the NF-κB pathway, cell cycle regulatory genes (*CDKN2A* and *RB1*), and immune evasion genes (*CD274* and *PDCD1LG2*) may be pathologically associated with IPT-like FDCS (14).

The morphology of IPT-like FDCS is similar to that of the conventional type. Gross examination reveals that most tumors exhibit a well-marginated, thin-walled, yellowish, soft tissue mass (maximum diameter = 7.54 ± 4.93 cm). In particular, localized hemorrhage or necrosis is observed within the tumor. The neoplastic cells, which may exhibit mild atypia, are usually spindle, ovoid, or polygonal and form storiform, fascicles, or trabecular arrays, which exhibit sparsely vesicular chromatin and distinct nucleoli (15). In particular, the inflammatory component of IPT-like FDCS, a more prominent histology, comprises mainly lymphocytes (B and T cells), plasma cells, eosinophils, and rare epithelioid histiocytes, with neoplastic cells often obscured by the inflammatory infiltration (6, 16). Owing to the lack of atypical tumor cells, IPT-like FDCS are often incorrectly identified inflammatory-reactive processes or inflammatory pseudotumor, even other various neoplasms (17). Moreover, the scarcity of cases and lack of specific clinical and imaging features present a formidable challenge to diagnosing IPT-like FDCS. Currently, the diagnosis of IPT-like FDCS requires auxiliary tests, including imaging, detecting distinctive cytological features, immunohistochemical detection of FDC markers, and *in situ* hybridization to detect EBER.

Although limited reports are available on the imaging features of IPT-like FDCS, they aid in making correct diagnoses before treatment when a neoplasm is detected (18). Most unenhanced computed tomography (CT) images display circular or elliptical, slightly hypodense tumors with a clear boundary. In certain cases, significant necrosis is seen within the tumor, while calcification or hemorrhage is rare. The lesions typically show heterogeneous enhancement in the enhanced phase, although the enhancement state is lower than that of the parenchyma. Therefore, the tumor area is always hypodense compared with the periparenchyma, and annular enhancement is observed in the delayed phase, sparing the central necrotic region (19). MRI and CT images are similar, and most lesions demonstrate enhancement from the center to the periphery in the arterial phase. The enhancement amplitudes of lesions in the portal, venous, and delayed phases tend to be homogeneous and diminished to varying degrees, and annular enhancements are occasionally observed (20).

The diagnosis of IPT-like FDCS is invariably supported by immunohistochemistry, and multiple FDC markers are often necessary, including CD21, CD23, CD35, CXCL-13, D2-40, Clusterin, Fascin, epidermal growth factor receptor, and CNA42 (21, 22). In particular, CD21 and CD35 are the most specific with almost universal positivity (23). Nevertheless, some EBV-related IPT-manifesting lesions do not express FDC markers (16). The immunohistochemical analysis of SSTR2a in FDCS indicates a positive rate significantly higher than CD21 and CD35 in conventional subtypes, while all IPT-like variants are negative (24). Therefore, SSTR2a shows promise as a highly sensitive and differential diagnostic marker to distinguish between FDCS and IPT-like FDCS. As mentioned previously, IPT-like FDCS is closely associated with EBV infection, while conventional types infrequently involve EBV (12). Our literature review identified only five cases of intrabdominal, EBER-negative IPT-like FDCS (4, 9, 25–27). In our present case, immunohistochemical analysis of the pathological specimen detected strongly positive expression of CD21, CD35, Ki67 (> 20%), and EBER (Figure 2).

Inflammatory pseudotumor (IPT)-like FDCS is a low-grade malignant tumor with good prognosis. Unlike FDCS, IPT-like FDCS is apparently indolent, with few instances of recurrence and metastasis (3). Disease status at the time of last follow-up is known for 92 patients, with follow-up times ranging from 2 to 144 months. Only 9.65% of patients (*n* = 11) experienced recurrence or metastasis during follow-up. Yet, PNP-associated IPT-like FDCS predominantly occurs in intraabdominal sites, indicating poor prognosis (8–10). Surgery is the most effective therapy for IPT-like FDCS, and only two cases (Cases 49 and 63) received chemotherapy or targeted therapy. However, chemotherapy, radiotherapy, or targeted therapy do not achieve a significant improvement in overall-or disease-free survival (28). Notably, Cases 49, 90, and 104 died because of multiple organ dysfunction syndrome, cachexia, and disseminated tumor, respectively, during treatment. The possibility of recurrence and metastasis suggests conducting long surveillance after surgery.

## Conclusion

Inflammatory pseudotumor (IPT)-like FDCS is an extremely rare neoplasms that mainly occurs in the intraabdominal region. EBV probably plays an essential role in the etiology of IPT-like FDCS. The diagnosis of IPT-like FDCS is complex and usually relies on fine-needle aspiration biopsy or postoperative pathological diagnosis. Surgical resection is the most effective treatment, although the efficacy and safety of adjuvant chemotherapy, radiotherapy, or targeted therapy for postoperative management are unknown. IPT-like FDCS presents a certain risk of recurrence or metastasis after initial treatment. Thus, regular follow-up visits are strongly recommended.

## Data availability statement

The raw data supporting the conclusions of this article will be made available by the authors, without undue reservation.

## Ethics statement

The studies involving human participants were reviewed and approved by the Institutional Review Board review ([2021]12-114) at The Third Affiliated Hospital of Sun Yat-sen University in Guangzhou, China. The patients/participants provided their written informed consent to participate in this study.

## Author contributions

CX was the patient’s physician and responsible for the revision of the manuscript for important intellectual content. FD reviewed the literature and contributed to drafting the manuscript. CW performed the radiographic analysis. FD and HT conceptualized and designed the study, coordinated and supervised data collection, and critically reviewed the manuscript for important intellectual content. All authors issued final approval for the version to be submitted for publication.
